# The Successful Use of Bilateral 2-Level Ultrasound-Guided Stellate Ganglion Block to Improve Traumatic Brain Injury Symptoms: A Retrospective Analysis of 23 Patients

**DOI:** 10.1093/milmed/usae193

**Published:** 2024-05-17

**Authors:** Sean W Mulvaney, James H Lynch, Kristine L Rae Olmsted, Sanjay Mahadevan, Kyle J Dineen

**Affiliations:** Department of Military and Emergency Medicine, Uniformed Services University, Bethesda, MD 20814, USA; Department of Military and Emergency Medicine, Uniformed Services University, Bethesda, MD 20814, USA; RTI International, Research Park, NC 27709, USA; Regenerative Orthopedics and Sports Medicine, Annapolis, MD 21401, USA; Regenerative Orthopedics and Sports Medicine, Annapolis, MD 21401, USA

## Abstract

**Purpose:**

The purpose of the study was to determine whether performing ultrasound-guided, bilateral stellate ganglion blocks (SGBs; performed on subsequent days) improved traumatic brain injury (TBI) symptoms.

**Methods:**

A retrospective chart review was conducted for the time period between August 2022 and February 2023 to identify patients who received bilateral, 2-level (C6 and C4) SGBs for PTSD symptoms but who also had a history of TBI. Neurobehavioral Symptoms Inventory (NSI) scores were collected at baseline, 1 week, and 1 month post-treatment in 14 males and 9 females.

**Results:**

Out of 23 patients, 22 showed improvement in their NSI scores. NSI baseline average score was 42.7; the average score at 1 week post-treatment was 18.8; 1 month post-treatment was 20.1. This represents a 53% improvement in the NSI score between baseline and 1 month.

**Conclusion:**

The use of bilateral, 2-level SGBs may be indicated in treating patients with PTSD symptoms with concomitant diagnoses of mild-to-moderate TBI.

## INTRODUCTION

Traumatic brain injury (TBI) is a commonly reported injury among military personnel, with 15 to 20% of returning service members reporting having sustained a mild TBI.^1^ It is also extremely common in non-military populations, with 100 to 300 per 100,000 people seeking medical attention for mild TBI or up to 42 million people worldwide every year.^2^ In a study of soldiers returning from Iraq with TBI, 43.9% had concurrent PTSD.^3^ PTSD and TBI share many similarities in their pathophysiology. Both TBI and PTSD have been linked to physical trauma, although PTSD has also been correlated with emotional trauma.^4^ TBI and PTSD share both common symptoms and pathophysiology that perpetuate brain damage through neuroinflammatory, oxidative, and excitotoxic mechanisms.^5^ TBI and PTSD result in substantial brain morphology changes with abnormalities of the fronto-cingulo-parietal cognitive control network, which is involved in cognition, memory, attention, and inhibition of fear processing.^6^ Even if TBI/PTSD patients receive rehabilitation therapy, commonly offered therapy often affords palliative care and does not address the progressive nature of the disease. The chronic neuroinflammation may persist over time and exacerbate the behavioral abnormalities of TBI/PTSD.^7^

The stellate ganglion is a cluster of nerves and nerve fibers located in proximity to the C7 vertebra and plays a significant role in mediation of the sympathetic nervous system. The stellate ganglion block (SGB) is a treatment that has been shown to be effective in treating PTSD. There are currently nearly 30 publications in the peer-reviewed medical literature regarding SGB for the treatment of PTSD, which show safety and durable improvement in PTSD.

During routine follow-up on PTSD patients, we noted that in addition to the expected improvements in PTSD symptoms, both headaches and brain fog were clinically improving in our patients with a history of TBI. In response to that observation, in addition to our usual screening of follow-up with the Posttraumatic Stress Disorder Checklist for DSM-5 (PCL-5)^8^ and Generalized Anxiety Disorders Version 7 (GAD-7),^9^ we started to include the Neurobehavioral Symptom Inventory (NSI) in patients with a previous diagnosis of chronic TBI. Although not intended to diagnose TBI, the NSI has value for clinicians and researchers in characterizing the presence and severity of symptom complaints and tracking symptomatic change in persons with TBI.^10^ The NSI has been adopted by the U.S. DoD and the DVA for both research and clinical evaluation of TBI.^10^

## METHODS

In all cases, the SGB was done for the treatment of PTSD and not for the purpose of treating TBI. Patients had a history of PTSD as established by previous diagnosis by a behavioral health provider and by a score of 40 on the PCL-5.^8^ The PCL-5 is a commonly used self-report assessment of PTSD symptoms with significant psychometric support.^11–14^Patients were identified for the time period August 2022 to February 2023 who had a concomitant previous diagnosis of mild/moderate TBI and who had completed the NSI at baseline, 1 week, and 1 month post-SGB. Those who scored over 14 on the NSI baseline score were included in the analysis.

The NSI questionnaires were filled out by the patients in person on paper and hand-keyed by clinic staff or emailed via a secure clinic email system. Follow-up NSI questionnaires (at 1 week and 1 month) were collected either via email or in person. This case series was approved by the institutional review board of the Institute of Regenerative and Cellular Medicine (IRCM-2023-358).

The 2-level SGBs were performed using techniques described in the medical literature.^15^ The ultrasound-guided 2-level cervical sympathetic chain blocks were performed using a 2-inch 25 gauge needle, using ultrasound guidance (General Electric Logiq e with a 8-12 MHz broadband linear transducer) by a lateral, in-plane approach, after a power Doppler scan and clear identification of the vertebral artery and vein and other vasculature, at both the sixth cervical vertebra level (using 6-8 mL of 0.5% ropivacaine) and the fourth cervical vertebra level (using 1.5-2 mL of 0.5% ropivacaine). The right-sided cervical sympathetic block was first done, with the left side done on the subsequent day to eliminate the risk of inadvertent bilateral inadvertent blockade of the recurrent laryngeal nerve and subsequent potential airway compromise. All procedures were performed at an established musculoskeletal practice by pain and sports medicine fellowship-trained physicians who have collectively performed more than 4,000 SGBs. The Horner’s syndrome, a sign of a successful blockade of the stellate ganglion, is characterized by ptosis, miosis, and scleral injection and was scored by 2 independent observers at 5 minutes post-block per published guidelines.^16^ All patients met the minimum clinical threshold for an acceptable Horner’s syndrome of 4 out of 6 points by both observers.

## RESULTS

**FIGURE 1.**
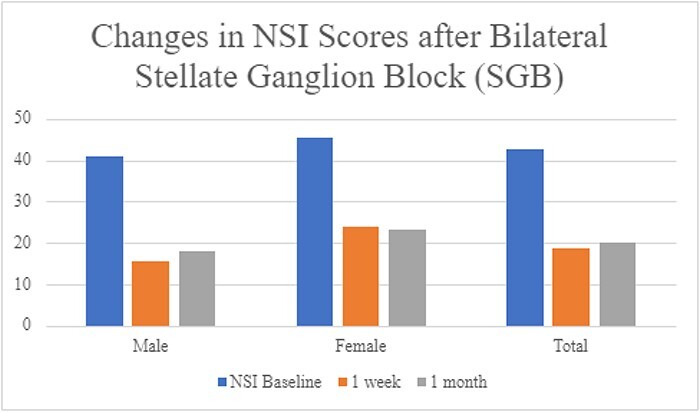
Changes in patient NSI scores following stellate ganglion block (SBG) intervention at baseline, 1 week and 1 month. Both male and female patients’ NSI scores decreased by nearly 50% following SBG intervention.

From August 2022 to February 2023, we compiled 23 completed patient records from patients with NSI scores at baseline, 1 week, and 1 month. Table I and Fig. 1 present descriptive data by gender for the sample. The results from SGB intervention on 23 patients (14 males and 9 females) showed marked improvements in NSI scores from baseline over the 1-week and 1-month follow-up periods. As depicted in TABLE II, the average improvement across all patients was over 55% after 1 week and 53% 1 month after intervention. Fig. 1 represents a marked decrease in NSI scores in all patients, from an average NSI baseline of 42.7 to 18.8 after 1 week, with a mild regression to 20.1 after 1 month.

**TABLE I.** Neurobehavioral Symptom Inventory (NSI) Questions. The Following Questions on the NSI Questions Are Each Scored on a 0 (No Symptom) to 5 (Severe Symptom) Scale

Feeling dizzy
Loss of balance
Poor coordination, clumsy
Headaches
Nausea
Vision problems, blurring, trouble seeing
Sensitivity to light
Hearing difficulty
Sensitivity to noise
Numbness or tingling on parts of the body
Change in taste and/or smell
Loss of appetite or increased appetite
Poor concentration, can’t pay attention, easily distracted
Forgetfulness, can’t remember things
Difficulty making decisions
Slowed thinking, difficulty getting organized, can’t finish things
Fatigue, loss of energy, getting tired easily
Difficulty falling or staying asleep
Feeling anxious or tense
Feeling depressed or sad
Irritability, easily annoyed
Poor frustration tolerance, feeling easily overwhelmed by things

## DISCUSSION

This is the first successful use of bilateral SGB to treat TBI symptoms. The primary findings of this study show the marked decrease in overall NSI values from baseline after the multi-level SGB intervention. The average NSI score improved by over 45% for both male and female patients following the first treatment, with both groups showing over 49% improvement 1 month after the multi-level SGB interventions. In the male treatment group, NSI scores improved by 56% from baseline 1 month removed from intervention, while the female group showed a 49% improvement from baseline.

**TABLE II.** Case Series Patient Demographics and NSI Scores at Baseline, 1 Week and 1 Month. Interval Improvements from Stellate Ganglion Block Intervention for Male and Female Patients Showing Improvements in NSI Score from Baseline at the 1-week and 1-month Mark

	Male	Female	Total
Number	14	9	23
Age range	35-55	23-55	23-55
Average age	44.1	39.2	42.2
NSI baseline score	40.9	45.6	42.7
NSI score: 1 week	15.6	23.9	18.8
NSI score: 1 month	18.1	23.3	20.1
Percent improvement: baseline to 1 week	61.89	47.56	55.91
Percent improvement: baseline to 1 month	56	49	53

We note a mild difference between male and female patients in their initial interval improvement 1 week removed from SGBs and their improvement over the 1-month follow-up period. The male group showed the highest improvement in NSI scores 1 week following SGB intervention, with an average improvement of 61.9%, before plateauing at a 56% improvement from baseline. This represents an interval change from an average NSI score of 40.9 to 15.6 after 1 week, then a marginal increase to 18.1 after 1 month. The female group showed a milder initial interval improvement of 47.6% 1 week after SGB intervention and then reported slight improvements over the 1-month period resulting in a 49% improvement from baseline. Female patient’s NSI scores averaged a baseline of 45.6, then decreased to 23.9 over 1 week, and then had further improvement to 23.3 after 1 month.

Although this is a small sample size, clinically, it is reasonable to consider male patients at a higher likelihood of showing a more robust initial response to SGB, while female patients may experience a more gradual improvement over the 1-month response period. This may also be reflective of female patients having a higher baseline NSI score values than males, as represented in Table I.

Yang et al. published their findings on improvement in plasma NF-κB and inflammatory factors in 25 patients treated with SGB for TBI versus 25 patients in their control group.^17^ Although their patient population was acute TBI, they stated that SGB might be involved in the regulation of the post-TBI nerve-endocrine-immune system dysfunction. Kim et al. showed that the unilateral SGB could increase the cerebral blood flow of this cerebral hemisphere.^18^ Finally, Ter Laan et al. published results to suggest that SGB’s mechanism of action on cerebral blood flow may be a promising therapy for what is believed to be dysfunctional cerebral perfusion and/or autoregulation, which can exist with a history of TBI.^19^ These studies suggest that there is the potential for dysfunctional cerebral perfusion and/or autoregulation to be re-set through the use of bilateral ultrasound-guided SGB procedures at the C6 and C4 levels.

In the first 2 case series describing SGB for the treatment of PTSD, the SGB was performed on the right side at the sixth cervical vertebra level.^20,21^ Initially, the right side was selected based on our understanding of the essential role of the right hemisphere in the human stress response.^22^ Per our 2015 Clinical Practice Guidelines publication, we began doing left-sided SGB only by exception if the right-sided SGB did not result in clinical benefit.^16^ In 2020, Rae Olmsted et al. published a multi-center randomized controlled trial, which showed the effectiveness of right-sided SGBs spaced 2 weeks apart for the treatment of PTSD symptoms.^23^ In the first reporting of the effectiveness of 2-level SGBs, our case series demonstrated that an ultrasound-guided 2-level SGB performed at both the fourth cervical vertebra level and the sixth cervical vertebra level resulted in superior results to an SGB at the sixth cervical vertebra alone.^15^ In this study, the fourth cervical vertebrae as the second level were selected for its relative safety and reproducibility under ultrasound guidance. In 2021, we published the first case series, which showed that 5% of patients with PTSD only responded to a left-sided SGB (versus a right-sided SGB).^24^ A more accurate term for a 2-level SGB is a 2-level cervical sympathetic chain blockade, since it does not specifically target the stellate ganglion, although SGB remains the commonly accepted term. In their 2023 article, Lynch et al. had a first reporting of not only durable improvements in anxiety, as measured by improvements in GAD7 scores, but also showed that bilateral SGB are both safe (when done on subsequent days) and may result in greater improvements in the GAD7 scores than a right-sided SGB alone.^25^

In 2021, we started to routinely incorporate bilateral 2-level SGBs for the treatment of PTSD. The right and then the left-sided 2-level SGBs were performed on subsequent days to eliminate the risk of inadvertent bilateral recurrent laryngeal nerve block and subsequent potential respiratory distress.

Our findings are limited by the following: It is a retrospective analysis, small sample size, and the lack of a formal TBI diagnosis requirement (other than the patient history and elevated NSI score). Another limitation is the fact that SGB already has fairly robust published evidence, which shows that it is beneficial in treating PTSD, and there is significant overlap in TBI and PTSD symptoms.

Our work demonstrates that the novel use of multi-level and bilateral SGBs may be an effective interventional treatment modality for persistent TBI-related symptoms.

## CONCLUSION

In this limited retrospective case series, the use of bilateral ultrasound-guided 2-level SGBs resulted in more than 49% improvement in NSI scores at 1 month post-treatment in both men and women with mild/moderate TBI with a concomitant diagnosis of PTSD. Further studies are needed to assess this treatment modality for the treatment of TBI.

## Data Availability

The data that support the findings of this study are available on request from the corresponding author. All data are freely accessible.
